# Population Fitness and the Regulation of *Escherichia coli* Genes by Bacterial Viruses

**DOI:** 10.1371/journal.pbio.0030229

**Published:** 2005-06-21

**Authors:** Ying Chen, Ido Golding, Satoshi Sawai, Ling Guo, Edward C Cox

**Affiliations:** **1**Department of Molecular Biology, Princeton University, Princeton, New Jersey, United States of America; Tufts University School of MedicineUnited States of America

## Abstract

Temperate bacteriophage parasitize their host by integrating into the host genome where they provide additional genetic information that confers higher fitness on the host bacterium by protecting it against invasion by other bacteriophage, by increasing serum resistance, and by coding for toxins and adhesion factors that help the parasitized bacterium invade or evade its host. Here we ask if a temperate phage can also regulate host genes. We find several different host functions that are down-regulated in lysogens. The *pckA* gene, required for gluconeogenesis in all living systems, is regulated directly by the principal repressor of many different temperate prophage, the cI protein. cI binds to the regulatory region of *pckA,* thereby shutting down *pckA* transcription. The *pckA* regulatory region has target sequences for many other temperate phage repressors, and thus we suggest that down-regulation of the host *pckA* pathway increases lysogen fitness by lowering the growth rate of lysogens in energy-poor environments, perhaps as an adaptive response to the host predation system or as an aspect of lysogeny that must be offset by down-regulating *pckA*.

## Abstract 2

Lysogenic bacteriophage such as lambda integrate into their host genome, but do they regulate specific host genes? This study shows that they do, thereby increasing the fitness of the lysogen.

## Introduction

A central question in the biology of host–parasite interactions is how a balance between the costs and benefits to both is achieved. If the burden to the host is too high, the parasite will go extinct, and for this reason it is often postulated that parasites confer some benefit upon their hosts, thereby arriving at an equilibrium in the competition between parasite-free and infected host populations.

**Figure 1:**
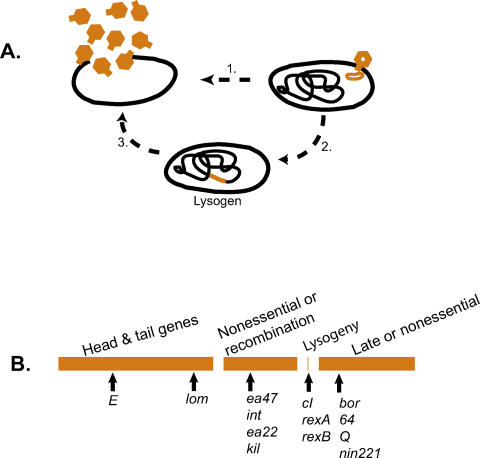
The λ Life Cycle and Gene Organization (A) The lytic compared to the lysogenic life cycle. Invading phage can either replicate and lyse the host cell (1), or they can integrate into the host genome (2) where they replicate as part of the host genome. At a frequency of approximately 10^−6^ to 10^−5^ per cell division, the prophage escapes cI repression, replicates, and lyses the host (3) (B) The genetic map showing the approximate organization of functional blocks and, below the map, the location of the genes up and down regulated as revealed in this study. The *lom* gene is embedded between two different tail-fiber genes. The genome length is 48.5 kb, approximately 75 genes. See [2,29].

Bacterial viruses are parasitic. Roughly speaking, they either invade and kill the host or they invade and lie dormant, either integrated into the host genome or outside it as extrachromosomal elements [1]. Phage that can exist in a dormant state are called temperate phage, and bacteria carrying temperate phage are said to be lysogenic. In the lysogenic state, viral functions needed for replication and packaging are shut down by a phage-encoded repressor. Occasionally, a temperate phage genome escapes repression, the virus begins to replicate, and soon the host cell lyses, producing a new generation of viral particles (Figure 1A). The lysogenic state thus imposes a cost on the bacterium because every so often the viral genome (prophage) replicates and kills the host. This selective disadvantage is offset, however, in several important ways. Because prophage produce a repressor that keeps the prophage genes from being expressed, the host bacterium enjoys immunity from lytic infection by temperate family members. A second, and different, mechanism confers immunity to lytic phage, those phage that cannot exist in the host as prophage [2,3]. Temperate phage may also confer fitness on a host by coding for genes that enhance host virulence and resistance to the immune system [4], of which there are dozens of examples: the Shiga toxin produced by some strains of *Escherichia coli;* the β toxin produced by *Corynebacterium diptheriae,* the causative agent of diphtheria; endotoxin production by *Clostridium botulinum;* staphylococcal endotoxins; and cholera toxin produced by *Vibrio cholerae,* to name a few. Then too, prophage often code for functions that allow the lysogen to successfully colonize the animal host [4], and in general, temperate phage increase horizontal gene flow in microbial populations [5]. Thus there are several advantages to being a lysogen, and at least one big disadvantage: Occasionally the prophage replicates and kills the host.

Of the many prophage that litter bacterial genomes, the best studied is λ. When it recombines into the *E. coli* chromosome, phage multiplication is shut down by the phage-encoded repressor, cI, a critical element of the genetic switch. The continuous low-level production of cI protects the host against further infection by extracellular λ, while also regulating the levels of cI synthesis intracellularly (reviewed in [6]). The phage also codes for genes required for replication, maintenance, integration, and escape from the host cytoplasm, as well as a series of genes not required for growth in the laboratory (Figure 1B).

Most λ genes are repressed by cI, but there are several whose transcription is constitutive, their expression either dependent or independent of cI control. Two, the products of the *rexA* and *rexB* genes, exclude productive infection by the unrelated lytic bacteriophage T2, T4, and T6 [2]. The Rex proteins have also been reported to increase the advantage of lysogens in competition experiments [7], but there are conflicting results in the literature on this point [8]. Two other proteins, Bor and Lom, are found in the host outer membrane, and *bor* lysogens are resistant to guinea pig serum [9,10]. Each of the above examples illustrates how prophage-encoded λ genes increase lysogen fitness by coding for a protein that protects the host from invaders or from the humoral system.

Here we ask if the phage repressor directly or indirectly regulates host genes. There is indirect evidence that a streptococcal temperate phage may regulate a bacterial gene that protects cells against phagocytosis [11], but there have been no systematic studies on this subject. By surveying both the host and phage genomes with microarrays, we have found several new and unexpected expression patterns in *E. coli* lysogens, in addition to those viral genes known to be expressed. One in particular, a host gene partly responsible for gluconeogenesis, *pckA,* is down-regulated many fold, and this leads to a growth disadvantage for the lysogen when grown on succinate, a common carbon and energy source. The DNA sequence lying upstream of the *pckA* coding region contains sequences homologous to the DNA-binding site for cI, and, surprisingly, for the cI homologs of other temperate phage. Thus it appears that down-regulation of *pckA* is part of an adaptive strategy for many different temperate phage.

## Results

### Expression Profiles

**Figure 2:**
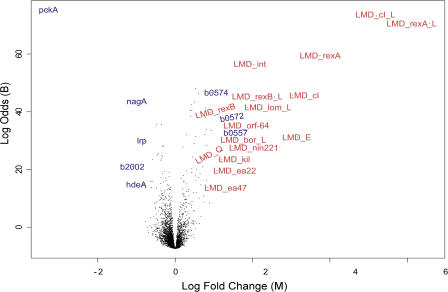
Gene Expression Pattern in Lysogen versus Nonlysogen In this representation, the log of the odds (B) [30] is plotted against the log of the ratio of the change (M) for each gene. The larger the odds, the higher the confidence in the fold change. M is log_2_(R_lysogen_/G_nonlysogen_), where R is the signal in the red channel and G the signal in the green. Twelve samples from each of two exponentially growing lysogen and nonlysogen cultures were harvested at 6-min intervals and used to prepare cDNA probes as described in Materials and Methods. cDNA samples from each time point were labeled separately with Cy3 and Cy5, mixed, and then used to probe the microarrays in duplicate. For each time point, Cy3 and Cy5 labels were reversed, and reversed labels were also used to probe the microarrays in duplicate. Thus each time point is the average of four datasets, and the data in Table 1 and Figure 2 represent 48 arrays probed with cDNA from exponentially growing cells. The dataset on which Table 1 is based may be found in supplemental Table S1.

Our arrays were designed to assay the expression levels of 4,539 *E. coli* and 73 λ open reading frames during exponential growth in tryptone broth, standard growth conditions for experiments with λ lysogens [2]. The results are summarized in Figure 2 and Table 1, in which we restrict our analysis to changes in gene expression levels of approximately 2-fold (|*log*_2_| levels of approximately one) or more.

**Table 1:**
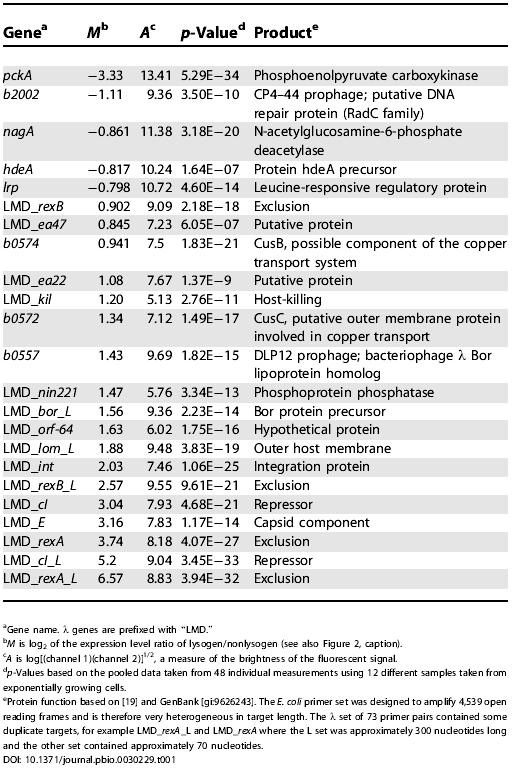
Host and Viral Genes Regulated in λ Lysogens

### Host Gene Expression in λ Lysogens: The *pckA* Gene

**Figure 3:**
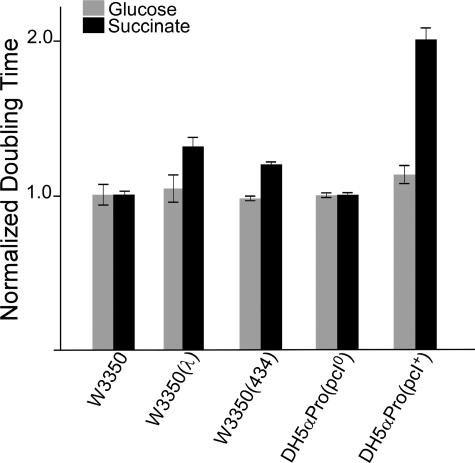
The Growth of Lysogens and Nonlysogens in Glucose and Succinate Media In this figure, pcI codes for cI, and pcI^0^ is the empty vector. See Materials and Methods for the genotypes of these strains. Growth rates were obtained by least-square fit of the optical density versus time curves. Error bars for the growth rates were obtained by fitting two subsets of the data and using the standard deviation of the two estimates.

The most striking change is in the expression level of *pckA,* the gene coding for phosphoenolpyruvate carboxykinase [12]. PckA in the presence of ATP converts oxaloacetate to phosphoenolpyruvate [13]. Mutant *pckA* strains grow less well on succinate and other carbon sources that feed into oxaloacetate [14], although phosphoenolpyruvate can also be synthesized from succinate via phosphoenolpyruvate synthase [13]. We expected that the down-regulation of *pckA* transcripts in lysogens might lead to a similar problem. Figure 3 compares the generation times of lysogens and nonlysogens of both λ and another temperate phage, λimm434, on glucose and on succinate as the sole carbon source (the λimm434 cI and operator sequences replace the λ homologs in λimm434; [15,16]). There is very little difference between lysogen and nonlysogen growth rates on glucose, but the difference on succinate is striking. In glucose, the difference in doubling time is at most a few percent, whereas on succinate it is increased at least 30% in λ lysogens and 20% in λimm434 lysogens. These are substantial changes in fitness: The lysogen will be reduced by 90% in 17 generations, 99% in 34 generations, and 99.9% in 50 generations when the doubling time differs by 20%.

**Figure 4:**
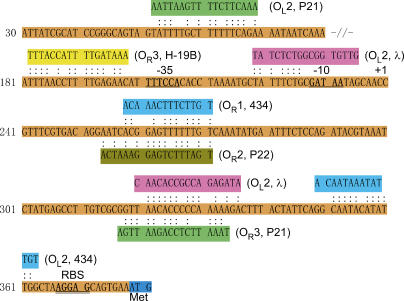
The Upstream *pckA* Region Homologous to Known Temperate Bacteriophage Operator Sites O_R_3, O_L_2, O_R_1, and O_R_2 are sequences known to bind either cI or the cI homologs of the lysogenic bacteriophage H-19B, λ, 434, P22, P21, and 434 shown in brackets next to each operator sequence [6,31,32]. A second P21 O_L_2 site lies further upstream from the −35 region.

In the *E. coli* genome there are many sequences that exhibit some homology to the λ operator [17,18]. The highest homology is with a 16/17-base pair (bp) match to O_L_2, and it occurs within the open reading frame of *rlpB,* a gene coding for an inner membrane lipoprotein. There are four additional sequences with a 15/17-bp match, two to O_R_1 and two to O_L_2. They are in the coding regions of *rlpB* and *pabC* for O_R_1 homologs, and *hmpA* and *yjgQ* for O_L_2 homologies, none of whose expression pattern is altered by our criteria. By relaxing our search for homologies to 11-mers, we find 244 more sites, but in all of these cases there are gaps in the matching strings. Some of these map in possible regulatory regions of the genome. The strongest λ homologies, however, map to the 370-bp sequence upstream of *pckA* (Figure 4).

There are homologies to other temperate phage operators in this region as well. Phages H-19B, 434, 21, and P22 are four such examples. The presence of these sequences suggests that the phenotypes of the two lysogens summarized in Figure 3 might be caused by cI binding to these related operator sites.

We first examined this idea by comparing the growth rates of strains carrying a cI-expressing plasmid to the parental strain carrying an empty plasmid (see Figure 3, DH5αPro(pcI) versus DH5αPro(pcI^0^)). There is a dramatic increase in doubling time when these strains are grown on succinate as the only carbon source (τ = 139 min vs. 278 min), and very little difference on glucose.

These results support the idea that cI expression depresses growth rate, but the data do not distinguish between direct and indirect effects. To make this distinction, we used real-time PCR to measure *lacZ* message in a strain where *lacZ* was fused to a 370-bp *pckA* upstream sequence (Table 2). Four different strains were compared: W3350 and W3350 (λ) as baseline controls (lines 1–4) and two different strains carrying *lacZ* on a plasmid (lines 5–10). One of these, DH5αPro(placZpcI), does not have a promoter and serves as a control on *lacZ* transcript levels. The other, DH5αPro(pP_pckA_lacZpcI), has the *lacZ* gene fused to a 370-bp upstream *pckA* sequence. Both strains also carried either *cI* on a plasmid, or the same plasmid lacking the *cI* gene.

**Table 2:**
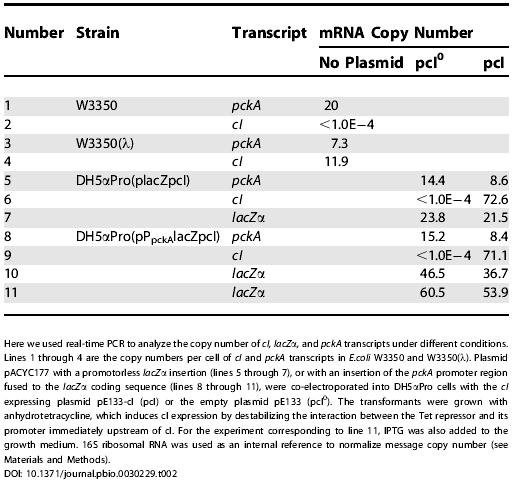
*lacZ* Is Regulated by cI When It Is Fused to the *pckA* Promoter Region

The chromosomal *pckA* transcript is down-regulated about 3-fold in lysogens compared to wild type (lines 1–4). Apparently cI acts directly on the *pckA* upstream sequence, because cI expression regulates *lacZ* transcript levels in strains where *lacZ* is fused to the *pckA* upstream sequence (lines 5–10). *cI* message is undetectable in nonlysogens, as expected (line 2), and the *pckA* transcript level is down-regulated 3-fold in a lysogen (line 1 compared to line 3). When cI is produced from a plasmid, it depresses *pckA* message approximately 2-fold (lines 5 and 8). In the *pckA* promoter-*lacZ* fusion, although the fold decrease in *lacZ* transcript level is not as large as it is in the previous examples, it is nonetheless down-regulated 25% to 30%. Because *lacZ* transcript levels are quite high in strains in which the *lacZ* gene lacks a promoter altogether (line 7), these smaller changes occur on top of a high background, possibly due to the high copy number of this plasmid. If we correct for this high background using these data, then the difference is more substantial, an approximately 35% reduction in transcript level in the strain carrying upstream *pckA* sequences. We note that even though the change in transcript number when *lacZ* is controlled by the *pckA* promoter region is not large, it is reproducible, and moreover, it is of the same order of magnitude as the changes in growth rate documented in Figure 2.

Taken together, these data suggest that cI binds to the upstream *pckA* sequence and down-regulates transcription. To define this interaction further, we asked if purified cI could bind to the *pckA* upstream sequence in a band shift assay. In these experiments, a 370-bp upstream region (Figure 4) was labeled with ^32^P using the polymerase chain reaction and incubated with affinity-purified cI protein. Samples were then analyzed by gel electrophoresis, and binding specificity was analyzed by competition with unlabeled DNA.

**Figure 5:**
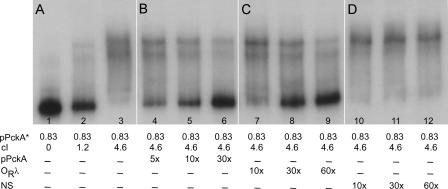
cI Binds to the *pckA* Promoter Region In these experiments, affinity-purified cI was added in variable (0 μM, 1.15 μM, and 4.6 μM, lanes 1, 2, and 3, respectively) or constant (4.6 μM, lanes 4–12) amounts to ^32^P-labeled upstream *pckA* DNA (pPckA*, 0.83 nM each lane). In lanes 4–5, a 5-, 10-, and 30-fold excess of unlabeled *pckA* DNA was added as competitor. In lanes 7–9, a 10-, 30-, and 60-fold excess of unlabeled λ O_R_ DNA was the competitor. In lanes 10–12, a 10-, 30-, and 60-fold excess of unlabeled random λ O_R_1 sequence (nonspecific) was added (see Materials and Methods).

Figure 5A shows that cI binds to the *pckA* DNA. The radioactive probe is authentic because it can be competed away with unlabeled upstream *pckA* DNA, as expected (Figure 5B). Moreover, unlabeled DNA containing λ operator sequences (O_R_λ) also competes, showing that our cI preparation responds to the authentic target DNA (Figure 5C). Finally, in the same assay, increasing quantities of a scrambled λ O_R_1 sequence fail to compete with cI binding, the expected result for a specific interaction between the promoter region lying upstream of *pckA* and the cI protein (Figure 5D).

Bacteriophage λ normally infects *E. coli,* and P22 infects *Salmonella*. Gluconeogenesis is an important metabolic pathway in most organisms [13], and so we have asked if the upstream *pckA* sequences are highly conserved in bacteria, or whether the cI–*pckA* interactions reported here are unique to the Enterobacteriaceae. Apparently, the *pckA* regulatory region is highly conserved in *Salmonella, Shigella,* and *Escherichia* species, but not elsewhere. These are all Enterobacteriaceae, suggesting that the cI–host interaction reported here is restricted to Gram negative bacteria (results not shown).

### Host Gene Expression in λ Lysogens: Other Genes

In addition to *pckA,* seven other host genes appear to be regulated approximately 2-fold in the lysogen (see Figure 2; Table 1). *b0557* is a *bor* homolog contained in the DLP12 prophage sequence resident in these strains [19]. Because it is 91% homologous to λ *bor,* this apparent increase in expression of a host gene is probably due to hybridization between λ prophage *bor* transcripts and the DLP12 resident prophage. We have not explored the significance of the six other transcript profiles shown here, other than to note that two up-regulated genes are copper transporters, and, like *bor* and *lom* [20], membrane proteins [19]. Finally, one of the down-regulated genes, *b2002,* is also embedded in another defective prophage, CP4–44, although it has no sequence homology with λ and is therefore likely to be directly or indirectly regulated by cI.

### *λ* Gene Expression

In general our results are consistent with the literature on λ gene expression patterns in lysogens (summarized in [2]). The promoter regulating *cI* expression is known to regulate the levels of *cI, rexA,* and *rexB,* as noted earlier, and our data clearly show elevated transcript levels. Likewise, both *lom* and *bor* are known to be transcribed in lysogens [9,20]. The Bor protein makes λ lysogens more resistant to guinea pig serum [9], and the Lom protein is involved in buccal cell adhesion in the gut [21]. Both are outer membrane proteins. *int* required for integration of the prophage into the genome, and *xis,* required for excision of the prophage from the genome when the prophage escapes repression, are expressed from the cI-independent promoters pL and pI and are active when the lytic/lysogenic decision is made and during early lytic multiplication [6]. Our data reveal, however, that *int* is also expressed in the lysogen, suggesting a possible strategy for stabilizing the prophage by kinetic means, in that elevated levels of Int protein might help stabilize the lysogenic state by shifting the equilibrium toward integration. This possibility would be in addition to the role that cI plays by repressing the functions needed for viral replication and packaging.

The product of the *E* gene is a procapsid protein essential for viral packaging. It is encoded in a polycistronic late message (see Figure 1), and is elevated at least 3-fold in the lysogen. *E* is part of a polycistronic message coding for genes needed for viral assembly, and thus finding elevated levels of *E* transcript is unexpected. However, in addition to cI repression of λ genes, it has been noted that there are strong Rho-independent transcription terminators flanking the *E* gene, and this has led to the idea that transcription terminators may add an extra level of prophage regulation, helping to silence gene expression by terminating aberrantly initiated polycistronic message production and translation [22]. Our finding of *E* up-regulation is consistent with this idea. In this context, it may also be significant that a similar termination sequence lies just downstream of *int.*

Finally, our microarray analysis suggests that five other λ open reading frames are also expressed in the lysogen: *orf-64, nin221, ea22, kil,* and*ea47*. Their transcripts are all elevated 2-fold or more. Whether or not there is a functional reason for this will require additional analysis.

In the microarray experiments, it may seem surprising that genes transcribed in the lysogen are not elevated many hundred- or even thousand-fold, because we are comparing ratios of samples with and without the λ genome. One has to remember, however, that background fluorescence is always present, and thus the reading is never zero, even from blank regions of the array.

## Discussion

Our results show that there is strong interaction between the host and parasite genome in this model system, and we can ask what role this interaction plays in the evolution of fitness in these populations.

We note first that lysogen growth rates on succinate are dramatically reduced both for λ and λimm434 lysogens (see Figure 3). These are very large fitness changes, in the 20% to 30% range per generation, a profound disadvantage for lysogens in a competitive environment in which succinate is the carbon source and, by extension, gluconeogenesis is important.

The second clear result is that there are at least seven DNA sequences with varying degrees of homology to the λ operator sites upstream of the *pckA* gene. These sites are homologous to operator sequences used by other temperate phage, and they lie either close to the –10 to –35 polymerase binding site, or between this region and the ribosomal binding site (see Figure 4). One, the O_L_2 homology of phage 21 may lie too far upstream to be considered part of the *pckA* regulatory domain. Were there only one or two λ-specific sequences in this region, we might conclude that they were there by chance alone. However, the many distinct potential cI binding sequences for H-19B, 434, 21, and P22 make the chance hypothesis unlikely. Note that these sites are dissimilar, reflecting the different target sequences for the different phage repressors—they are not simply differences within a single short consensus sequence. Although the λ O_L_2 sequence is at best a half operator, the others show substantial homology, and the 370-bp *pckA* sequence binds cI in vitro with an affinity at least as strong as the authentic λ consensus sequence (Figure 5).

The clustering of operator homologies suggests that there is strong selection pressure maintaining these sites and that they are an important aspect of lysogen fitness, one in which the regulation of a host gene, rather than the production of a phage product, confers increased fitness. There is currently insufficient knowledge about whole-genome *E. coli* expression patterns in different environments to speculate about what these selective pressures might be, but we suggest that lysogens may preferentially survive because of their lower growth rates in a glucose-poor environment—which would be the case, for example, if the immune system more effectively attacks rapidly growing cells in such an environment—or it could be that an aspect of lysogeny itself must be offset by down-regulating the *pckA* gene. Independent of the mechanism, our main conclusion is that the multiple potential operator sites lying upstream of the *pckA* gene suggest strong positive selection for this subtle host–parasite interaction, one that can be added to the known advantages of *cI* expression leading to immunity from superinfecting λ, protection from infection by T2 and T4 phage, and resistance to host serum factors.

How do these results compare to other studies with viral-infected cells? Following animal virus infection, many host genes are turned on and off. For example, in one early study of cells infected with human cytomegalovirus, 1,400 of the 12,626 genes surveyed changed by a factor of four or more following infection, and several of these changes pointed to the central role played by the immune system in a productive infection [23]. Other studies in a wide variety of infected cells confirm these general results—many host genes are both up- and down-regulated—but to our knowledge these changes in gene expression have not been firmly tied to phenotypic changes in host–virus dynamics.

## Materials and Methods

### 

#### Strains

W3350 is from the *E. coli* Genetic Stock Center (CGSC #5976) (http://cgsc.biology.yale.edu/, *galK2(Oc), galT22, LAM^-^,* and*IN(rrnD-rrnE)1*. DH5αPro is from Clontech (Palo Alto, California, United States), *deoR, endA1, gyrA96, hsdR17(r_k_^–^m_k_^+^), recA1, re/A1, supE44, thi-1, Δ(lacZYA-argF)U169, φ80δlacZΔM15, F^–^, λ^–^, P_N25_/tetR,* and *P_laci_^q^/laci, Sp^r^.* λ wild type is λW2 (λ PaPa), a gift from Dr. R. Weisberg, and λimm434 is bio16, a gift from Dr. D. Kaiser.

#### Microarray analysis

A total of 4,539 *E. coli* MG1655 open reading frames and 73 λ genes were amplified by PCR, purified by ethanol precipitation, and verified by gel electrophoresis. The *E. coli* MG1655 genome open reading frame primer set is from Sigma-Genosys (Woodlands, Texas, United States), and 73 primer pairs for λ genes were designed by using the Primer3 program (http://workbench.sdsc.edu) and synthesized by Integrated DNA Technologies (IDT; Coralville, Iowa, United States). A list of the genes and primers is available from the corresponding author. The purified PCR products were resuspended in 50% DMSO and printed onto Corning Ultra GAPS slides (Corning, New York, United States) using OmniGrid from Genomic Solutions (Ann Arbor, Michigan, United States). *E. coli* W3350 and W3350(λ) were grown in Tryptone medium with aeration, and 12 samples from each culture were taken about every 6 min between OD_600_ 0.1 and 0.6. RNA samples were extracted using the Qiagen RNeasy kit (Valencia, California, United States). RNA labeling and microarray hybridization were as described [24]. For each time point, W3350 cDNA samples were labeled with Cy3, and W3350(λ) cDNA samples were labeled with Cy5. The samples were mixed and used to probe the microarrays in duplicate. In addition, for each time point, Cy3- and Cy5-labeling schedules were reversed, and the reverse-labeled cDNA samples were also used to probe the microarrays in duplicate. Thus each time point is the average of four datasets, and the data in Table 1 and Figure 2 represent 48 arrays probed with cDNA from exponentially growing cells. The hybridized arrays were scanned using an Axon 4000B scanner (Sunnyvale, California, United States), and the data were analyzed using limmaGUI (http://bioinf.wehi.edu.au/limmaGUI/) with Bioconductor packages (http://www.bioconductor.org).

#### Bacterial doubling times

Strains were grown in M9 minimal media [25] supplemented with 1 μg/ml B12, and either 0.4% glucose or 0.4% succinate as the only carbon source.

#### Band shift assay

λ cI protein was purified using the PRO Tet 6xHN Bacterial Expression System (Clontech) as described [26]. The PCR product of the *pckA* upstream sequence (pPckA), which is the 370-bp *E. coli* genomic region 3530464 to 3530833 covering the *pckA* promoter region, was amplified using the primers TGGTTATCCAGAATCAAAAGGTG and GCTCCTTAGCCAATATGTATTGC, and labeled using α-P^32^-dCTP by PCR. Purified λ cI protein and labeled DNA were mixed in Sauer buffer [27] with or without competitors (pPckA, λ O_R_, or randomized λ O_R_1 DNA), incubated on ice for 2 h, and run on a precast 10% TBE native polyacrylamide gel from Bio-Rad (Hercules, California, United States). Gels were dried and exposed to x-ray films. The competitor λ O_R_ was amplified by PCR using primers CGTCCTCAAGCTGCTCTTGT and GCGCATTGCATAATCTTTCA, and is 184 bp long. Another competitor, randomized λ O_R_1, was synthesized by IDT and is 17 bp long.

#### Real-time PCR analysis

The λ cI expression vector pE133-cI (“pcI” in the text) was constructed by cloning the *cI* gene (coordinates 37227 to 37940 on the λ genome) into the expression vector pPROTet.E133 (Clontech). The *E. coli* *pckA* upstream sequence fused to the *lacZα* coding sequence, the *lacZ* promoter sequence followed by the *lacZα* coding sequence, or the *lacZα* coding sequence alone were cloned into the *Sac*II/*Nhe*I sites of pACYC177. The pE133 vector or the pE133-cI vector and each of the three modified pACYC177 vectors were electroporated together into DH5αPro electro-competent cells (Clontech). The transformed strains were grown under different conditions, depending on the experiment. RNA samples were extracted using Qiagen RNeasy kits. *cI, lacZα,* and *pckA* message numbers were analyzed by real-time PCR, using the SYBR Green PCR Master Mix from Applied Biosystems (Foster City, California, United States). 16S ribosomal RNA was used as a reference to normalize message copy numbers, assuming 20,000 copies of the rRNA molecule per cell [28]. Primers TGCATCTAGAGGGCCCAATTC and CGGGCCTCTTCGCTATTACG were used to detect *lacZα,* primers GCATAA CGTCGCAAGACCAA and GCCTAGGTGAGCCGTTACCC were used to detect 16S rRNA, primers ATGCGCGTTAACAATGGTTTGA and TAGTTAACACCCCGCGCTCAT were used to detect *pckA,* and primers GTTGAAGGTAATTCCATGACC and ACTAGCGATAACTTTCCCCACA were used to detect *cI*.

## Supporting Information

Table S1 Host and Viral Genes Regulated in λ Lysogens Columns A, B, C, and D are the spot coordinates; Column E: The *E. coli* gene ID number of the original ORFmer primer set; Column F: gene name (λ genes are prefixed with “LMD”); Column G: *M* = log_2_ of the expression level ratio of lysogen to nonlysogen (see also Figure 2 caption); Column H: *A* = log [(channel 1)(channel 2)]^1/2^, a measure of the brightness of the fluorescent signal; Columns I, J, and K: Statistic based on the pooled data from 48 individual measurements using 12 different samples taken from exponentially growing cells. The *E. coli* MG1655 primer set from Sigma-Genosys (Woodlands, Texas, United States) was designed to amplify 4,539 open reading frames and is therefore very heterogeneous in target length. The λ set of 73 primer pairs contained some duplicate targets, for example, LMD__*rexA*__L and LMD__*rexA* in which the L set was approximately 300 nucleotides long and the other set contained approximately 70 nucleotides. The LMD primer set is available from the corresponding author (E-mail: ecox@princeton.edu.
(764 KB XLS).

### Accession Numbers

The GenBank (http://www.ncbi.nlm.nih.gov/Genbank/) accession numbers for the genes and gene products discussed in this paper are *b0557* (GeneID 948980), *b2002* (GeneID 946514), *cI* (GeneID 1238683), *E* (GeneID 2703482), *ea22* (GeneID 2703469), *ea47* (GeneID 2703536), *hmpA* (GeneID 947018),*int* (GeneID 2703470),*kil* (GeneID 2703471), *nin221* (GeneID 2703476), *orf-64* (GeneID 2703478),*pabC* (GeneID 946647), *pckA* (GeneID 945667), *rexA* (GeneID 2703537), *rexB* (GeneID 2703517), *rlpB* (GeneID 946257), *xis* (GeneID 2703504), *yjgQ* (GeneID 1037318), and λ genes (NC 001416)*.* The GenBank accession number for the *E. coli* MG1655 primer set is U00096.

## Abbreviation

bp: base pair
